# Carbonic Anhydrase Inhibitors Induce Ferroptosis through Inhibition of AKT/FTH1 Signaling in Ewing Sarcoma Tumor Cells

**DOI:** 10.3390/cancers15215225

**Published:** 2023-10-31

**Authors:** Darya Fayzullina, Semyon Yakushov, Kamilla Kantserova, Elizaveta Belyaeva, Denis Aniskin, Sergey Tsibulnikov, Nafisa Fayzullina, Stanislav Kalinin, Olga Romantsova, Peter S. Timashev, Brett A. Schroeder, Ilya V. Ulasov

**Affiliations:** 1 Group of Experimental Biotherapy and Diagnostics, Institute for Regenerative Medicine, World-Class Research Centre “Digital Biodesign and Personalized Healthcare”, I.M. Sechenov First Moscow State Medical University, 119991 Moscow, Russia; dfaizullina@yandex.ru (D.F.); sem.yakushov@gmail.com (S.Y.); kam11_03@mail.ru (K.K.); belyaevaliza2000@gmail.com (E.B.); denaniskin@yandex.ru (D.A.); ser-tsibulnikov@yandex.ru (S.T.); 2 Institute for Regenerative Medicine, Sechenov First Moscow State Medical University (Sechenov University), 119991 Moscow, Russia; nafisa.fayzullina@gmail.com; 3 Department of Chemistry, Saint-Petersburg State University, 199034 Saint-Petersburg, Russia; s.kalinin@spbu.ru; 4 Research Institute of Pediatric Oncology and Hematology at N.N. Blokhin National Medical Research Center of Oncology, Ministry of Health of Russia, 115478 Moscow, Russia; dr.roma1986@gmail.com; 5 World-Class Research Centre “Digital Biodesign and Personalized Healthcare”, I.M. Sechenov First Moscow State Medical University, 119991 Moscow, Russia; 6 National Cancer Institute, Center for Cancer Research, Bethesda, MD 20814, USA; brett.schroeder@nih.gov

**Keywords:** Ewing sarcoma, toxicity, carbonic anhydrase, fibroblasts

## Abstract

**Simple Summary:**

Ewing sarcoma (ES) is one of the most common kinds of cancer in youngsters, and ES relapse after therapy treatment remains a problem. Carbonic anhydrases (CAs; EC 4.2.1.1) have emerged as interesting molecular targets for the development of anticancer medicines as a fundamental regulator of cellular homeostasis. In this investigation, we used the commercially available acetazolamide and previously found CA inhibitors to target the CAII isoform, which was overexpressed and positively linked with relapse in ES patients. We detected that CAII inhibitors can trigger ferroptosis in ES cells by downregulating FTH1 and increasing cathepsin B expression, inhibit cell growth, limit invasion, and trigger apoptosis or autophagy-related cell death. The findings imply that cytosolic CAII may be a promising target for ES therapy, and CAII inhibitors may be useful as single-agent or combination antitumor therapies in the treatment of ES.

**Abstract:**

Ewing sarcoma (ES) is one of the most frequent types of malignant tumors among children. The active metabolic state of ES cells presents a new potential target for therapeutic interventions. As a primary regulator of cellular homeostasis, carbonic anhydrases (CAs; EC 4.2.1.1) have emerged as promising molecular targets for the development of anticancer drugs. Within the present study, we tested the commercial drug acetazolamide and our previously discovered inhibitors to target the CAII isoform, which was overexpressed and positively correlated with ES patient relapse. We employed molecular biology tests to identify effective inhibitors of CAII that can induce ferroptosis by downregulating FTH1 expression in ES cells. In vitro, we have also demonstrated their ability to reduce cell proliferation, decrease invasion, and induce apoptosis- or autophagy-related cell death. Using Western blotting, we confirmed the induction of cathepsin B in cells treated with CA inhibitors. It was found that the suppression of cathepsin B expression during the treatment reduces the anticancer efficacy of selected CAII inhibitors. These experiments highlighted profound antitumor activity of CAII inhibitors attributive to their remarkable ability to trigger ferroptosis in Ewing sarcoma cells without causing substantial host damage. The obtained results suggest that cytosolic CAII may be a prospective target for ES treatment, and CAII inhibitors can be considered as potential single-agent or combination antitumor agents to be used in the treatment of ES.

## 1. Introduction

Cancer remains a major global cause of mortality, persisting despite extensive efforts to combat it. According to the World Health Organization, malignant tumors were the leading cause of premature death in 2019 in many developed countries. Globally, cancer ranked as the second-leading cause of death, trailing only cardiovascular disease [1]. Despite the discovery of novel treatments, the implementation of maintenance therapies, and the innovation and incorporation of new medications and procedures into treatment regimens, cancer mortality continues to rise.

The treatment of Ewing sarcoma in particular is a significant challenge for medicine. According to different statistics and classifications, Ewing sarcoma is an aggressive tumor of mesenchymal origin that accounts for 10% to 15% of all bone sarcomas [2]. The highest occurrence is observed during the second decade of life, primarily affecting teenagers [3]. There has been a substantial breakthrough in the treatment of localized malignancies during the last 40 years, with five-year survival rates improving from 20% to 70% [4].

ES relapse is a common type of ES recurrence. Recurrence rates, however, remain high (25%), and there is no conventional therapy for recurrent Ewing sarcoma [5]. Elevated levels of IGF-1R, IGF-1, IGFBP-1, and IGFBP-3 [6], APBA1 and NLGN4X [7] or SOX2 [8] are independent predictors of ES patients to relapse. The hallmark of ES cells is activity of multiple metabolic indicators, which suggests high metabolic activity [9]. The most common manifestation of tumor cells with metabolic activity is activity of cellar genes responsible for glucose uptake [10], extracellular matrix metabolism in general and heparan sulfate proteoglycan catabolism [11], including activity of enzymes involved in oxidative metabolism [12] in response to therapy. A recent study by Wang et al. [13] implicated activity of carbonic anhydrase in osteosarcoma resistance and protection from stress. Now, it is known that a hypoxic environment raises the possibility of sarcoma cells becoming functional [14,15] due to cellular enzymes such as carbonic anhydrases, which control cellular homeostasis. Carbonic anhydrase is a family of zinc metalloenzymes that comprises 15 isoforms exhibiting various subcellular localizations and catalytic activities. Some of these isoforms are implicated in the maintenance of the sarcoma phenotype. Thus, the CAIX isoform has been reported to play a vital role in malignant cells’ adaptation to hypoxia and acidosis in multiple tumor types. Moreover, several studies demonstrated that aberrant expression of particular CA isoforms, including the cytosolic CAII and transmembrane CAIX isozymes, may provide prognostic value for patients with soft-tissue sarcoma [16,17,18]; however, to the best of our knowledge, there are no data on whether CA inhibition would be beneficial for the therapy of Ewing sarcoma cells under normoxic conditions.

In our current study, we explored the potential of both the commercial pan-isoform CA inhibitor acetazolamide (AAZ) and a series of CA-inhibitory small molecules developed in-house, referred to as Inh1–10 [19,20,21,22], in the context of sarcoma treatment (for structures and K_i_ values against some therapeutically relevant CA isoforms, see SI, Table S1). Interestingly, we found that AAZ actually enhances invasion and vimentin expression in Ewing sarcoma tumor cells. To delve deeper into the anticancer effects of CA inhibitors in ES cells, we conducted transcriptome sequencing. Our research involved various tests, including MTT, Alamar blue, and clonogenic assays, which led us to identify several inhibitors with significant anticancer efficacy against primary patient-derived ES tumor cells. Subsequent investigations revealed that these inhibitors have the potential to reduce clonogenicity or tumor cell growth while also inducing ferroptosis mediated by FTH1, without triggering apoptosis or autophagy.

## 2. Methods

### 2.1. Cells and Reagents

Primary patient-derived Ewing sarcoma cells ES30, ES33 and ES36 were developed using a protocol described earlier [10] (Supplemental Figure S1). Established primary cell cultures ES36, ES33 and ES33 have been characterized via PCR (to detect EWS/FLI1 fusion) via immunofluorescence (to detect CD99 and SOX2 cellular markers) and have been authenticated using multiplex PCR of 22 loci with STR repeats (SBT-RealGene SCREEN, SystemBiotech, Moscow, Russia). A673 cells were obtained from Sergey Boychuk (KFU, Kazan, Tatarstan Republic, Russia). Doxorubicin-resistant ES36 cells were prepared as described earlier [10]. All cells were cultured in RPMI 1640 medium (Paneco, Moscow, Russia) containing 10% fetal bovine serum (Cat # F800821, Intl Kang, Global Kang Biotechnology, Beijing, China) and 1% penicillin–streptomycin (Kino Co., Ltd., Hangzhou, China) in a humidified atmosphere containing 5% CO_2_/95% air at 37 °C. The antibodies were obtained from Servicebio, FineTest, and Cusabio (all Wuhan, China) as well from Dako (Agilent Technologies, Santa Clara, CA, USA). The specific catalogue numbers and working dilutions are specified for each assay and antibody in the method section.

Acetazolamide (AAZ, pan-Ca inhibitor) and RSL3 (inhibitor of glutathione peroxidase 4 (GPX4) (ferroptosis activator)) were provided by Dr. S. Kalinin (St. Petersburg State University) and Dr. M. Conrad (Pirogov Russian National Research Medical University, Laboratory of Experimental Oncology, 117997 Moscow, Russia), respectively. Z-VAD-FMK and tamoxifen were purchased from Selleck Chemicals (Houston, TX, USA) and Sigma (St. Louis, MO, USA). Carbonic anhydrase inhibitors (TAS19 (renamed Inh1), TAS21 (Inh2), TAS22 (Inh3), OX-71 (Inh4), OX-72 (Inh5), OX-73 (Inh6), OX-74 (Inh7), Py-05 (Inh8), Py-23 (In9) and Py-26 (Inh10)) were provided by Mikhail Krasavin and Stanislav Kalinin (Department of Chemistry, Saint Petersburg State University, Saint Petersburg, Russia) (Table S1). For the purpose of simplicity these inhibitors were renamed to Inh1, Inh2…Inh10. A mixture of siRNA duplexes against human CTSB was purchased from Santa Cruz Biotechnology (Dallas, TX, USA).

### 2.2. Single-Cell RNA-Seq and Data Analysis

The data of single-cell RNA sequencing of patient-derived Ewing sarcoma tumor cells (ES36) and human embryonic fibroblasts (M19) were obtained from Yakushov et al. [10] study. The single-cell RNA-sequencing data of 46 ES patients were obtained from a Children Oncology Group’s study (GSE63155). We performed standard analysis procedures.

### 2.3. Bioinformatic Analysis

The GEO-based dataset GSE63155 was analyzed to show a connection between carbonic anhydrase isoform mRNA expression in ES tumors and vital status of ES patients over a course of 5 years after therapy, to build Kaplan–Meier overall survival curves for prognostic significance of CAII for ES patients, and to demonstrate an association of CAII with cellular proteins. Briefly, to establish a link between CAs tumor expression and ES patient survival, for each detected CA, the ES patients were divided into two groups based on survival status. Expression of each CA in alive vs. non-alive patient group was averaged and statistically analyzed. For Kaplan–Meier survival analysis, ES patients were categorized into high and low expression, then grouped based on median CAII gene expression value. Finally, for Gene Ontology analysis, around 200 of the most differentially regulated genes were detected with respect to survival status. Based on Mann–Whitney analyses (*p* < 0.05), we selected 116 genes with the most differential expression between the CAII-positive and CaII-negative tumor tissues. Using string analyses (www.string-db.org), the top 50 genes were plotted to demonstrate their interaction with each other and involvement in biological processes (Gene Ontology). Possible associations were demonstrated for 20 cellular proteins based on String software (STRING: functional protein association networks (www.string-db.org)).

### 2.4. Alamar Blue Cell Viability Assay

The cell viability assay was performed using adherent cells. Briefly, 10,000 cells/well were seeded in a 96-well plate overnight and then exposed to AAZ, Inh1–10, or DMSO for 72 h. At selected time points, the culture medium was replaced with 10% AB solution (in the culture medium Alamar blue cell viability agent (Cat # DAL1100, Invitrogen, Eugene, OR, USA) that was added to each well. Four hours later, 100 μL aliquots were transferred into a black opaque 96-well plate (Thermo Fisher Scientific, Waltham, MA, USA). The fluorescence intensities of the cell medium supernatant incubated with AB solution were measured at 544 nm excitation and 590 nm emission using a 1420 Victor3 multilabel plate reader (Perkin Elmer, Waltham, MA, USA). The fold of mock growth was calculated as suggested by the vendor.

### 2.5. Clonogenic Assay

ES36 and A673 cells were cultured for 2–3 days in standard conditions (medium supplemented with 10% FBS) prior to spheroid formation. To assess the clonogenic capacity of drug-treated cells, cells were trypsinized and resuspended in growth media to a final concentration of 4 × 10^6^ cells/mL and plated in agarose nonadhesive 12-well plates created using 3D petri dish molds (Microtissues, Providence, RI, USA). The agarose plates with cell suspensions were rested for 1 h and then completely covered with growth media. In 3 days, the cultured spheroids (37 °C, 95% humidity, 5% CO_2_) received growth media with appropriate drug concentration. After 3 and 7 days, the numbers of colonies were counted using phase contrast microscopy (×10). Clonogenic efficiency was determined as the average number of colonies per dish for each treatment group based on 10 fields per treatment.

### 2.6. Scratch Assay

Migration of wounded A673 cells in the presence of carbonic anhydrase inhibitors was assessed by performing an in vitro scratch assay in which a linear wound midline was performed across the bottom of the dish on a confluent monolayer of epithelial cells using a 200 µL sterile pipette tip. After that, cells were rinsed gently with PBS to remove any remaining cell debris. Complete RPMI medium was used during the scratch assay. DMSO or carbonic anhydrase inhibitors Inh4, 5, 6 or 9 at a dose of 100 μM were used as mentioned in the Methods section. Micrographs of the cells were taken at 4× magnification using a microscope equipped with a Moticam 10 digital camera at 72 h after beginning the experiment. Epithelial cell migration across the wound line was then quantified using Image J software (ImageJ (nih.gov)) by measuring the scratched area filled with cells in the mock-, Inh4-, 5-, 6- or 9-treated culture dishes.

### 2.7. MTT

Cancer cells or M19 were seeded (10,000 cells/well) in a 96-well plate in complete medium. After 12 h, cells were treated with either AAZ or carbonic anhydrase inhibitors (Inh1–10) at concentrations of 100, 10, and 1 μM for 3 days. The percentage of mock proliferated cells was assessed by MTT reagent assay following the manufacturer’s instructions. The highest concentration of DMSO was used as the vehicle control. Fold of mock growth cells vs. the experimental group is reported as the mean ±standard deviation.

### 2.8. LDH Assay

For determination of tumor cell lysis, the release of lactate dehydrogenase (LDH) in the cellular coculture supernatants was measured by a Pierce LDH Cytotoxicity assay kit (Cat#88954, Thermofisher Scientific, Meridian Rd., Rockoford, IL, USA), as recommended by the vendor.

### 2.9. Immunofluorescence

Immunofluorescence staining was performed using primary and secondary antibodies: anti-vimentin (Dako, CA, USA), secondary antibody, and chicken anti-rabbit (AlexaFluor 488 (H + L), Invitrogen, Grand Island, NY, USA). Images were acquired and analyzed using Image J software and all files were saved in JPEG format.

### 2.10. Flow Cytometry

Annexin V-AF488 (Lumiprobe Rus, Moscow, Russia) was used to analyze the apoptosis level of Ewing sarcoma cells treated with AAZ or carbonic anhydrase inhibitors 4, 5, 6, or 9 for 72 h. PI was employed to mark live tumor cells. After treatment, cells were harvested and transferred to a 15 mL tube. Antibodies were added at a ratio of 1:500 and incubated at room temperature for 10 min in the dark. Then, 10,000 cells were analyzed. Data were gathered as dot plot densitograms based on triplicate measurements and then plotted as a bar diagram. Average early or late apoptotic cells were presented.

### 2.11. Western Blotting

Cells were lysed according to a method described previously to obtain processed protein [23]. We separated the processed proteins by SDS-PAGE and transferred them to a PVDF membrane (Millipore, Darmstadt, Germany). After the PVDF membrane was blocked with 5% nonfat dry milk in PBS Tween-20, the membrane was successively incubated with the diluted primary antibody solution and the secondary antibody (Proteintech, Wuhan, China). The antibodies anti-BAX (Cat. No. FNab10410, 1:1000), anti-Beclin1 (Cat. No. FNab10873, 1:2000), anti-ATG5 (Cat. No. FNab00678, 1:1000), anti-cathepsin B (Cat. No. FNab10389, 1:1000), anti-CaII (Cat. No. FNab01161, 1:1000), and anti-phosphoAKT (Cat. No. FNab06402, 1:2000) were purchased from FineTest (Wuhan, China). Anti-FTH1 (Cat. No. CSB-PA008485, 1:1000), anti-Ca12 (Cat. No. CSB-PA0601116, 1:1000), anti-Ca1 (Cat. No. CSB-PA004364ESR1HU, 1:1000), and anti-Ca10 (Cat. No. CSB-PA878879EA01HU, 1:1000) were provided by Cusabio Technology (Wuhan, China). Anti-GAPDH (Cat. No. GB15002, 1:2000) and anti-LC3 (Cat. No. GB11124, 1:2000) were purchased from Servicebio (Wuhan, China). After primary antibody incubation, membranes were washed three times with PBS Tween-20 and then incubated with secondary antibodies conjugated with horseradish peroxidase (goat anti-rabbit, ab205718, 1:20,000 and goat anti-mouse, ab205719, 1:20,000). Finally, membranes were washed and protein bands were visualized using ChemiDoc Touch (BioRad, Hercules, CA, USA) to capture images. The relative protein level was normalized to GAPDH.

### 2.12. RNAi Knockdown

A673 and ES36 cells grown in 96-well plates (10,000 cells per well) or 6-well plates (200,000 cells per well) were transfected with a pool of scramble siRNA (Sranmble siRNA) or short interfering RNAs (Santa Cruz Biotechnology, sc-29238, USA) targeting the human CTSB gene using lipofectamine RNAiMAX reagent (Invitrogen, 13778-075) following the manufacturer’s instructions.

### 2.13. H&E and Immunohistochemistry of Patient Tumor Sections

The 9 Ewing sarcoma tissues were collected from patients with Ewing sarcoma at N.N. Blokhin National Medical Research Center of Oncology, Ministry of Health of Russia and provided by Dr. O. Romantsova. H&E-stained slides from each FFPE block were evaluated by two pathologists. Prior to staining, the working dilution for CAI (Cusabio, Cat # CSB-PA004364ESR1HU, 1/200), CAII (FineTest, Cat #FNab01161, 1/200), CAVIII (Cusabio, Cat # CSB-PA004379ESR2HU, 1/100), CAX (Cusabio, Cat # CSB-PA878879EA01HU, 1/400) and CA12 (Cusabio, Cat # CSB-PA060116, 1/50) antibodies was assessed using tumor sections of human renal cell adenocarcinoma primary tissue embedded in paraffin. The selected tissues were stained with primary antibodies followed by goat anti-rabbit IgG H&L (HRP) (Abcam, ab205718, 1:20,000, Boston, MA, USA). The scoring of positive cells in 10 HPF (high-power field) magnification ×400 was conducted independently by two certified pathologists representing Sechenov First Moscow State Medical University.

### 2.14. Statistical Analysis

Graphing and statistical analysis were performed using Prism GraphPad 6.0. Unpaired Student’s *t*-test and ANOVA were used to test for significance, and in all analyses, significance was taken as *p* < 0.05. All experiments were performed in triplicate or with six replicates per condition.

## 3. Results

### 3.1. CA2 Gene Elevates Expression in Primary ES Cells

**Figure 1:**
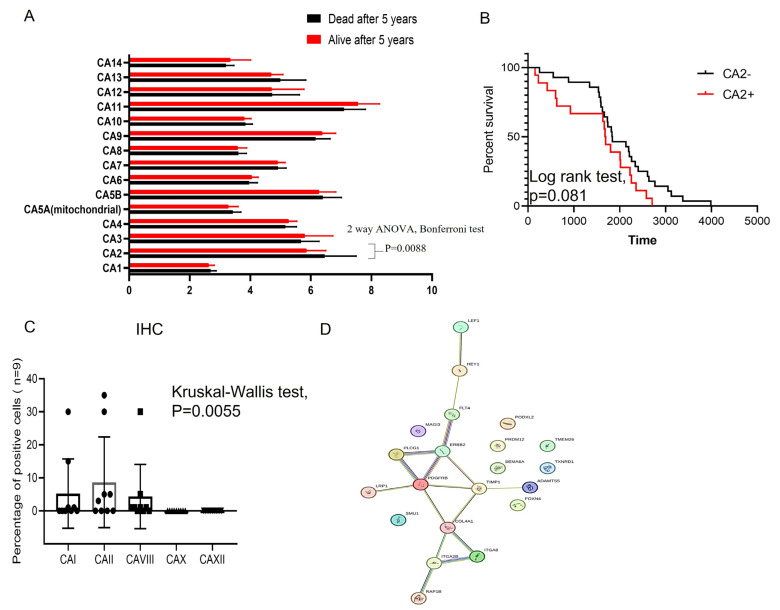
Identification of CA-mRNAs in the tissue of ES patients. (**A**) Box plots show the level of CA-mRNA expression in the ES tumor tissue of patients with relapse (red bar) and no relapse (black bar). Two-way ANOVA, Bonferroni test, *p* = 0.0088 for CA2 mRNA; (**B**) Kaplan–Meier plots for overall survival of the patients with ES using the GSE63155 dataset. Patients were classified according to CA2 mRNA expression (as described in Methods section). Log-rank test *p* value 0.081; (**C**) Box plots show the level of CAI, CAII, CAVIII, CAX and CAXII protein expression using primary paraffin-embedded ES tumor sections (N = 9) via immunohistochemistry. Kruskal–Wallis test *p* = 0.0031; PPI network constructed using STRING software (STRING: functional protein association networks (www.string-db.org)). for the 50 most differentially expressed and top21 (**D**) proteins exhibiting highest degree of correlation with CAII (Spearman degree of correlation between 7.2 × 10^14^ till 5.2 × 10^14^) were used.

We initially assessed the significance of carbonic anhydrase (CA) mRNAs in patients with Ewing sarcoma, both with and without relapse, using the GSE63155 dataset. This dataset contained mRNA sequences from 46 Ewing sarcoma patients, divided into two groups based on high and low expressions of Cas (based on median gene expression values). Among the detected CA mRNAs, high expression of CA2 mRNA was found to be directly correlated with patient relapse (Figure 1A, 2-way ANOVA, Bonferroni correction, *p* = 0.0088). To assess the clinical significance of this finding, survival curves were generated (Figure 1B). Although the log-rank test showed a marginal significance for gene expression (*p* = 0.081), we further validated this finding using primary Ewing sarcoma patient samples.

Our data indicate that among the five tested CA proteins, CAII exhibited significantly elevated levels in nine randomly selected primary samples with Ewing sarcoma (Supplemental Figure S2; Figure 1C, Kruskal–Wallis test, *p* = 0.0055). To delve deeper into the molecular pattern associated with CAII protein expression, we analyzed the GSE63155 dataset and identified the expression of 200 genes (Table S2). Among these, 116 displayed significant differential expression in CAII-positive Ewing sarcoma tumors (Mann–Whitney test, *p* < 0.05). Furthermore, Gene Ontology-based analysis of the 50 most regulated genes in CAII-positive Ewing sarcoma tissue (using String software) is shown in Figure 1D. This analysis suggests a direct association of CAII with genes involved in angiogenesis (GO: 0001525, FDR 2.3 × 10^−5^, and GO: 0001568, FDR 1.8 × 10^−8^) and cellular migration (GO: 0010632, FDR 0.00022, and GO: 0030334, FDR 0.00045). These findings suggest that the inhibition of CAII gene expression and/or enzymatic activity could offer therapeutic benefits for patients with Ewing sarcoma.

### 3.2. Inhibition of CAII Decreases ES Tumor Cell Proliferation

Expression analysis of ES-associated signature genes and molecules involved in disease-associated signaling pathways has previously been conducted [24,25,26]. To assess the potential impact of inhibiting CAII-mediated pathways on ES tumor proliferation and migration, we employed several low- to sub-nanomolar CAII inhibitors. In vitro experiments were conducted using Ewing sarcoma patient-derived ES30, ES33, and ES36 tumor cells to evaluate the effectiveness of each inhibitor. Human embryonic fibroblasts (M19) served as normal cell controls. For comparison of anticancer activity, we included acetazolamide (AAZ), recognized for its inhibition of CAII, IV, VII, and XII expression [27], and cisplatin, known for its anticancer properties in blocking osteosarcoma invasion [28].

**Figure 2:**
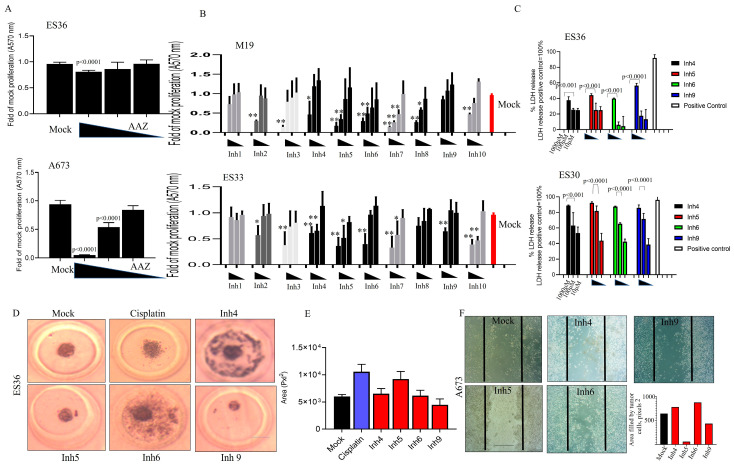
Blocking carbonic anhydrase impacts the proliferation of 2D-grown and spheroid growth of Ewing sarcoma cells. Cell proliferation of A673, patient-derived primary (ES36, ES33, and ES30) Ewing sarcoma cells, and embryonic M19 fibroblasts was assessed using the Alamar blue test or LDH. AAZ (**A**) and Inh1–10 carbonic anhydrase inhibitors (**B**,**C**) with various Kd (Table S1) at doses of 1000, 100, and 10 μM were added to the cells to evaluate cellular sensitivity (**A**,**B**) or cellular toxicity (**C**, LDH test) mediated by drugs 72 h after treatment began. Each experiment was performed with six replicates per treatment, and a multiple *t* test (**A**–**C**) was used to compare cellular proliferation with respect to drug concentration. Mock proliferated cells represent red bard. Results are shown as average ± SD; Statistical difference was presented vs. mock treated: “**” *p* < 0.001 and “*” *p* < 0.05; ES36 patient-derived Ewing sarcoma cell growth in the presence of cisplatin or inhibitors of cellular carbonic anhydrases (Inh4,5, 6, or 9) at a dose of 100 μM. The formation of ES36-based spheroids (**D**,**F**) or cellular motility/invasion (**F**) was monitored using a light microscope for up to 3 days (Scale 300 μm, magnification 100×). The volume of spheroids per treatment was calculated using Image J software and plotted based on 10 measurements per treatment (**E**). Cisplatin was used as a positive control. The Kruskal–Wallis statistical test was applied. Results are shown as average ± SD. (**F**) Wound healing scratch assay was performed on 24 h growth confluent A673 cells by scratching a line across the bottom of the culture dish. Inhibitors 4, 5, 6 and 9 or DMSO were added to the culture media and the cell motility and migration was observed at 72 h. The micrographs show the extent of scratch closure obtained under control conditions compared to those with the addition of inhibitors. Cell migration was evaluated by measuring the width in pixels between the cellular edges. Overall, 10 randomly selected images were collected, scratched area filled by cells was calculated, and data are presented as average of scratched area filled by cells.

The data presented in Figure 2A reveal that in contrast to AAZ, inhibitors Inh 4, 5, 6, and 9 consistently inhibited the proliferation of 2D-cultured ES30, ES33, and ES36 cells in a dose-dependent manner (Figure 2B). Notably, LDH detection demonstrated a significant increase in the number of LDH-positive ES cells during Inh 4, 5, 6, and 9 treatments, and this trend was dose-dependent (Figure 2C). The results from the colony formation assay (Figure 2D,E) showed a reduction in colony numbers in ES36-treated cells upon treatment with Inh 6 and 9. Additionally, Inh 9-mediated therapy effectively inhibited the motility and invasion of A673 cells (Figure 2F).

### 3.3. Inhibition of CA Expression Leads to Activation of Apoptotic and Autophagic Protein Expressions

**Figure 3:**
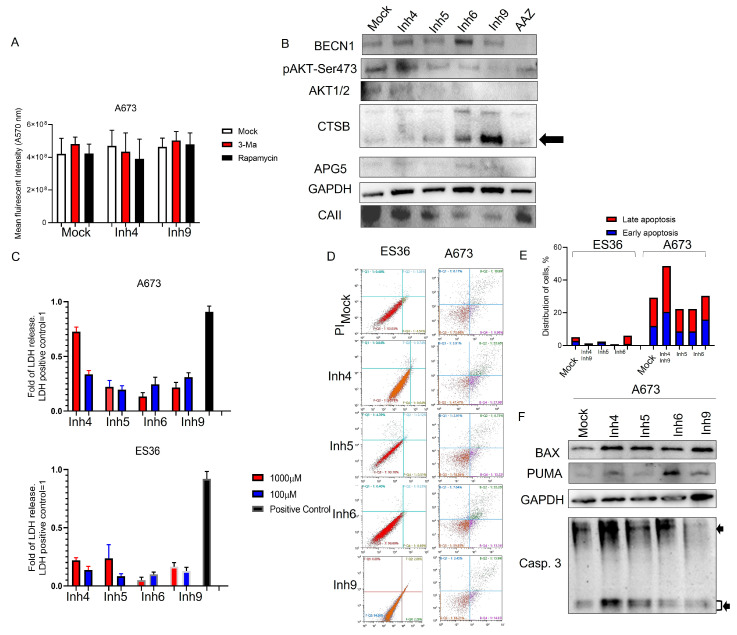
Cellular proliferation mediated by carbonic anhydrases is independent of apoptosis and autophagy signaling pathways. A673 and patient-derived ES36 were pretreated with 3 Ma (5 mM), rapamycin (10 mM) for 2 h or mock-treated in the presence of 100 μM of Inh4, Inh5, Inh6, or Inh9. After 48 (**A**,**B**) or 72 (**C**–**F**) h incubation, cells were analyzed via Alamar blue (**A**), LDH (**C**), stained with annexin V (**D**,**E**) via flow cytometry, or collected and then lysed in RIPA buffer (**B**,**F**). Thirty micrograms of total protein lysates were probed with antibodies against BAX, GAPDH, cleaved caspase 3, Beclin1, PUMA, carbonic anhydrase II, cathepsin B, Akt1/2, phospho-AKT and APG5. Arrows at subfigure B point to the accumulation of cathepsin B in Inhibitor-treated cells and arrows at subfigure D point to the expression of processed and unprocessed form of caspase 3 during inhibitor treatment. MTT and LDH assays were performed with 6 replicates per treatment during two independent measurements. Data are presented as average ± SD. Data from annexin staining were plotted as bar diagrams based on two replicates per condition. Overall, distribution of the A673- and ES36-treated cells during apoptosis is presented. The uncropped blots are shown in Figure S4.

To clarify the mechanism underlying the inhibition of ES proliferation and colony formation, we investigated whether programmed cell death played a role. Initially, we treated A673 and ES36-patient-derived ES cells with 100 μM of Inh 4 or Inh 9, along with autophagy suppressor (3Ma) or inducer (rapamycin). After 72 h, cellular proliferation was assessed using the Alamar blue assay. As depicted in Figure 3A, the addition of 3 Ma or rapamycin did not significantly change the effect of Inh 4 and Inh 9, suggesting that autophagy does not contribute to the observed cell death during treatment with these compounds.

Furthermore, we conducted Western blotting analysis of cell lysates from A673 cells treated with 100 μM inhibitors. We observed that the Ca inhibitors produced differential pattern expressions for BECN1. As shown in Figure 3B, Inh6 and Inh4 induced more Becn1 than AAZ-, Inh9-, Inh5-, and mock-treated cells. At the same time, a slight increase in APG5 and a decrease in phosphorylated AKT-Ser473 proteins were detected in the cells treated with Inh6 and Inh9. These two inhibitors also showed strong cathepsin B accumulation in ES cells, which negatively correlated with CAII protein expression in cells, suggesting a relationship between CTSB and CAII during cell treatment with Inh4, Inh5, Inh6, or Inh9.

To rule out apoptosis as the primary cause of ES cell death during treatment with carbonic anhydrase inhibitors, we performed several assays. LDH analysis (Figure 3C) showed an increase in LDH release in the CA-treated groups. Annexin V–PI staining (Figure 3D,E) and staining with antibodies against PUMA, BAX or cleaved caspase 3 (Figure 3F) were used to assess apoptosis. The results revealed that the proportion of LDH-release and ES cells labeled with annexin V–PI in the CA treatment group increased (late apoptosis up to 10–20% compared to the mock-treated control group for most of the inhibitors). To further support these findings, we measured the expression of apoptosis-related proteins, PUMA, BAX and cleaved forms of caspase 3 in the treated cells via Western blotting. The results indicated that the proapoptotic proteins PUMA and BAX showed a slight increase in cells treated with Inh4, 6, and 9, while the proapoptotic protein cleaved caspase 3 was detected only in the presence of Inh4. Therefore, our results suggest that Inh4 might induce some apoptosis, but the low percentage of apoptotic cells and the absence of autophagy induction point to some alternative mechanism of cell death mediated by the CA inhibitors in ES cells.

### 3.4. CAII Suppression Promotes Ferroptosis at ES Cells

**Figure 4:**
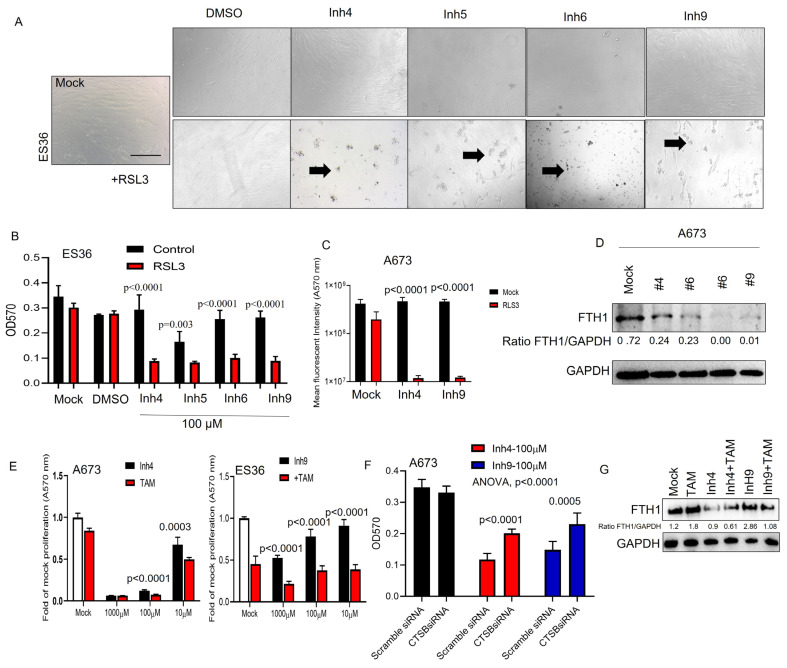
Decreased carbonic anhydrase cellular expression results in non-apoptotic cell death, such as ferroptosis. (**A**) Morphology of patient-derived Ewing sarcoma ES36 cells during different drug treatments. Upper panels: phase-contrast micrographs of cancer cells grown in a 2D monolayer and then treated with DMSO, inhibitors of carbonic anhydrases 4, 6, 7, and 9 (upper panel) or during additional RSL3 treatment (lower panel) comparison of cytotoxic effects mediated by carbonic anhydrase inhibitors in the presence of RSL3 using MTT (**B**) and Alamar blue (**C**) tests. Arrows picked up separated death cells. Scale bar 100 μm. Two independent experiments with six replicates per condition were used. Kruskal–Wallis values vs. carbonic anhydrase-treated cells alone are presented. (**E**,**G**) Tamoxifen enhances ferroptosis during inhibition of cellular carbonic anhydrases in Ewing sarcoma cells. Results of tumor cells cotreated with TAM (2 μM) and/or Inh4, Inh9 are shown as average ± SD. (**D**,**G**) WB analysis of the FTH1/GAPDH ratio in A673 (*t* test, TAM-treated vs. mock-treated, *p* < 0.0001 and 0.0003) and ES36 in the presence of mock or tamoxifen treatment upon 48 and 72 h of treatment (*t* test vs. untreated). The representative images of Western blot are shown. (**F**) Blocking cathepsin B decreases the anticancer activity of inhibitors for carbonic anhydrase Inh4 and Inh9. A673 cells were transfected with Scramble or CTSB siRNAs prior to treatment with 100 μM of Inh4 or Inh9. MTT analysis was performed 72 h later, and data are plotted as mean ± SD. Kruskal–Wallis statistical test vs. samples treated with Inh4 or Inh9 alone. The uncropped blots are shown in Figure S5.

It has been reported that the suppression of carbonic anhydrases may increase apoptosis along with the induction of ferroptosis [29,30,31,32,33]. To determine whether ferroptotic cell death was activated under treatment with selected CA inhibitors, we assayed the proliferation and cellular viability rates of ES36 and A673 cells. As shown in Figure 4A–C, the addition of ferroptosis-triggering RSL3 significantly enhances the reduction of cellular proliferation (40–60% increase vs. Inh4, 5, 6, or 9 alone treatment) and also induces more cell death than each of the inhibitors used (Figure 4C). On the molecular level, selected CA inhibitors reduced FTH1 expression by 40–90% compared to mock-treated A673 cells (Figure 4D). Earlier, Huang et al. [34] demonstrated enhanced acidification in tumor cells upon tamoxifen (TAM) release from tumor-targeting nanoparticles. Considering that inhibition of CA also disrupts cellular compartmentalization, the addition of TAM might enhance ferroptosis and cell death mediated by CA inhibitors. As seen in Figure 4E, cotreatment of A673 with various doses of CA inhibitors (1000, 100 and 10 μM) in the presence of TAM (2 μM) improves the cytotoxicity of cancer cells. Moreover, inhibition of cathepsin B mRNA expression (Figure 4F) during CA inhibitor treatment or the addition of TAM reduces overall toxicity and improves ferroptosis (Figure 4G), suggesting a cathepsin B-dependent mechanism of ferroptotic cell death during the application of CA inhibitors.

## 4. Discussion

The findings of our study provide new insight on metabolic traits in ES cells and protein signaling that contributes to the tumor’s aggressiveness. The obtained results demonstrate that ES cells heavily rely on cellular enzymes to control malignant phenotypes via a CAII-dependent mechanism. We observed that alteration of CAII-mediated pathways makes ES cells very vulnerable to ferroptosis-inducing drugs like RSL3. Our discovery that the CAII-based tumor cell phenotype is closely associated with ferroptosis susceptibility is significant, because it suggests that the metabolic trait of ES malignancies might offer a possibility for new therapeutic interventions. Importantly, we demonstrated that inhibiting CAII causes cell death in ES cells, suggesting that this pathway is essential for ES survival. Decreased CAII expression levels proved to correlate with better outcomes in ES patients, suggesting that this pathway is also important for tumor progression in clinical settings.

Further studies are of utmost importance to gain a better understanding of how ES cells utilize CAII-dependent metabolism. Given the previously observed reduction in milieu acidification by CAII in malignant sarcoma cells using AAZ [35], it is likely that higher expression of various CAs is required for survival, as they play a critical role in cellular bioenergetics in a hypoxic environment. Additionally, it has been reported that the expression of CAs may depend on the activity of the PI3K signaling pathway, which regulates the activity of CA promoters during oxygen deprivation [36]. Similar effects may occur when reducing stress through a PI3K modulator. Interestingly, previous research by Wang et al. demonstrated that the downregulation of another CA isoform, CAVIII, in human osteosarcoma cells sensitized tumor cells to apoptotic stress induced by staurosporine and cisplatin [13]. Depletion of CAVIII resulted in decreased phosphorylation of AKT, leading to a stress response characterized by reduced cellular invasion and colony formation. This insightful precedent supports the role of CAs in sarcoma homeostasis, even though, being catalytically inactive, CAVIII is not considered a target for competitive inhibitors [37,38]. Our findings demonstrate that CAII inhibition induces vulnerability in ES tumors by activating proapoptotic proteins like caspase 3, while also reducing stress-induced ferritin heavy chain 1 (FTH1). This makes tumor cells treated with CA inhibitors extremely sensitive to ferroptosis inducers such as RSL3, which showed enhanced toxicity, and tamoxifen, which also impacts the metabolic state of tumors via regulation of acidification [34].

Curiously, Inh4 and Inh9 differentially affected tumor homeostasis and cellular invasion. This suggests the impact of additional factors, such as the compounds’ metabolic stability, membrane permeability, off-target binding and/or potential involvement of additional targets, including other CA isoforms. While the specific molecular basis impacted by CAII inhibition remains to be identified, our results suggest that cellular vimentin may be one of the affected downstream targets. In fact, considering the high degree of vimentin protein expression in primary ES cells (Supplemental Figure S3) and the direct Pearson correlation between CAII and vimentin, we suggest that ES cells may rely on vimentin for adhesion and tumor proliferation. This hypothesis can be further supplemented by similar findings previously reported for other cancer cell types [39,40]. Overall, our research indicates that the accumulation of an acidic metabolic state due to treatment with CA inhibitors may render ES cells vulnerable to ferroptosis inducers, especially in the presence of FTH1 modulators [30,41,42]. These findings align with previous studies that have shown a connection between acidification and ferroptosis promotion [43] (graph caption), as well as the modulation of CA expression changing the threshold for ferroptosis-inducing treatments like radiotherapy [33,41].

## 5. Conclusions

Our research findings suggest that the changes induced by CA in ES cells could be targeted therapeutically through various approaches. CAII in particular seems to play a crucial role in regulating ES cell proliferation and invasion. Inhibiting CAII has been shown to be cytotoxic to ES cells, indicating that specifically targeting CAII could be a potential treatment strategy. In summary, the maintenance of altered pH and the growth of ES tumors are closely interconnected, and the development of medications that can target both of these pathways holds significant potential for the treatment of progressive diseases like Ewing sarcoma.

## Data Availability

Please address all inquiries regarding chemical inhibitors to Stanislav Kalinin (Saint Petersburg State University) and regarding cells to Ilya Ulasov (Sechenov University, Moscow).

## Supplementary Materials

The following supporting information can be downloaded at: https://www.mdpi.com/article/10.3390/cancers15215225/s1, Supplementary File S1: Figure S1. Microscopic analysis of Ewing sarcoma primary cultures ES36, ES33 and ES30. Figure S2. Detection of Carbonic anhydrase type II in the Ewing sarcoma tumor tissue. Figure S3. CA II inhibition manifests vimentin reduction in ES sarcoma cells. Table S1. Structure and Ki of several inhibitors against hCAI, hCAII, hCA IX and hCAXII. Table S2. List of genes most differently regulated in Alive vs. nonalive ES’ patient tumor samples 8 (GEO-based dataset GSE63155. Supplementary File S2: Figure S4: The original blots for Figure 3. Figure S5: The original blots for Figure 4.

## Abbreviations

AAZ	Acetazolamide
APBA1	Amyloid beta precursor protein-binding family A member 1
AKT1	AKT serine/threonine kinase 1
BAX	BCL2-associated X, apoptosis regulator
CAII	Carbonic anhydrase isoform II
DMSO	Dimethyl sulfoxide
GAPDH	Glyceraldehyde-3-Phosphate Dehydrogenase
IGF-1R	Insulin-like growth factor 1 receptor
IGF-1	Insulin-like growth factor 1
IGFBP-1	Insulin-like growth factor binding protein 1
IGFBP-3	Insulin-like growth factor binding protein 3
FTH1	Ferritin heavy chain 1
LDH	Lactate dehydrogenase A
NLGN4X	Neuroligin 4 X-linked
ES	Ewing sarcoma
PI3K	Phosphoinositide 3-kinase
PUMA	BH3-containing protein
SOX2	SRY-box transcription factor 2
TAM	Tamoxifen
