# Supplementary material for: Carbonic Anhydrase Inhibitors Induce Ferroptosis through Inhibition of AKT/FTH1 Signaling in Ewing Sarcoma Tumor Cells
Source: Cancers (Basel). 2023 Oct 31;15(21):5225. doi: 10.3390/cancers15215225 (PMC10650537; doi:10.3390/cancers15215225)
Supplement: Supplementary file 1 [file cancers-15-05225-s001.zip › supplementary file S1.pdf]

Table S1. Structure and Ki of several inhibitors against hCAI, hCAII, hCA IX and hCAXII.

ID	Structure	Ki, nM			
		hCA I	hCA II	hCA IX	hCA XII
**Inh1**	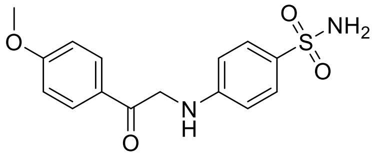	124.50	38.60	10.10	81.70
Inh2	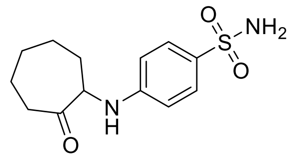	202.30	91.60	6.40	49.20
Inh3	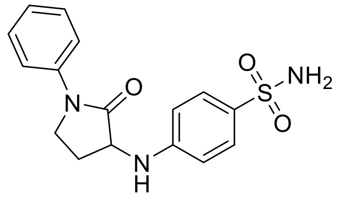	62.00	36.90	9.50	24.90
Inh4	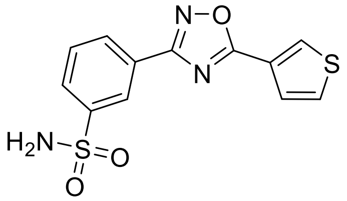	82.80	0.38	0.88	9.40
Inh5	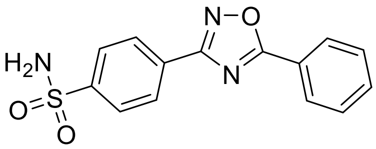	69.40	0.74	0.77	7.80
Inh6	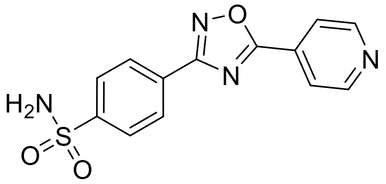	48.00	0.30	0.44	8.20
Inh7	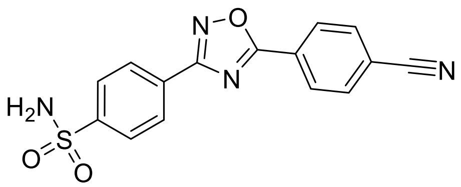	88.20	0.44	0.60	8.50

Inh7	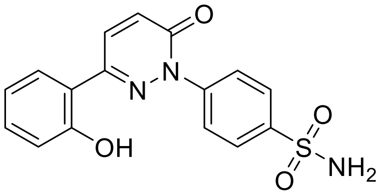	84.10	0.62	0.46	9.30
Inh9	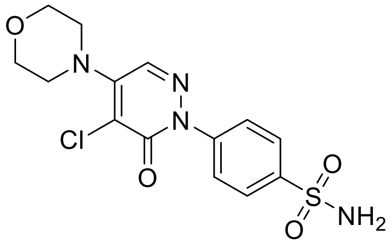	89.00	0.07	0.58	36.00
Inh10	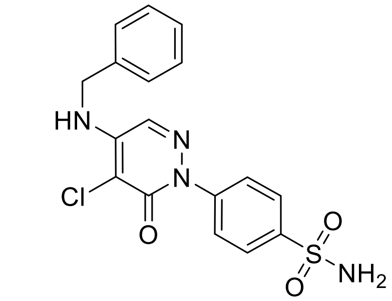	95.10	0.09	0.67	9.40

Table S2. List of genes most differently regulated in Alive vs nonalive ES′ patient tumor samples (GEO-based dataset GSE63155. Spearman correlation p values obtained in correlation between 200 cellular genes and CAII mRNA expression in ES patient tumor tissue. The results are significant for genes with p value less than 0.05 (Genes are selected in bold).

GENE	p value
**PLVAP**	0.000112
**KDR**	0.000133
**RFTN1**	0.000259
**PLXNA2**	0.000305
**FGD5**	0.000358
**RASGRP3**	0.00042
**DLL4**	0.000531
**PLXNA2**	0.000779
**PLEKHO2**	0.001216
**GPR116**	0.001307
**PTPRB**	0.001405
**ST8SIA4**	0.001405
**TM4SF18**	0.001405
**BMP8A**	0.001405
**CXorf36**	0.001509
**CD34**	0.001509
**GUCY1B3**	0.001509
**HPCAL1**	0.001621
**CD93**	0.001621
**VEGFC**	0.00174
**RAB27B**	0.001866
**ESAM**	0.002001
**COL4A2**	0.002001
**ADAMTS9**	0.002145
**STC1**	0.002298
**ENG**	0.002635
**GIMAP8**	0.002635

**ITGA9**	0.002819
**A2M**	0.003015
**MYCT1**	0.003444
**CDH5**	0.003678
**MMRN2**	0.004191
**ST3GAL6**	0.004191
**ROBO4**	0.00447
**INSIG1**	0.00447
**TSPAN15**	0.004766
**HEY2**	0.004766
**CD68**	0.005079
**TGFBR2**	0.005761
**SOX13**	0.005761
**CD276**	0.006131
**PCDH12**	0.006131
**HCLS1**	0.006131
**ARAP3**	0.006936
**PDGFB**	0.006936
**TP53I11**	0.007833
**RUNX1T1**	0.008318
**FILIP1**	0.008318
**PEA15**	0.00883
**GRB10**	0.009369
**LPL**	0.009937
**RAPGEF5**	0.009937
**ANPEP**	0.009937
**SPP1**	0.009937
**GIMAP6**	0.009937
**NCEH1**	0.009937
**CD200**	0.010534
**LYN**	0.011162
**TIE1**	0.011162
**LAPTM5**	0.012518
**KITLG**	0.012518
**CCR1**	0.013247
**ELMO1**	0.013247
**TMEM8A**	0.013247
**PGF**	0.014013
**FLT4**	0.014013
**PODXL**	0.014817
**RASGRF2**	0.014817
**TMEM204**	0.01566
**PRSS23**	0.01566
**RNF128**	0.016544
**SH2D3C**	0.017471
**DCBLD1**	0.017471
**PPIC**	0.017471
**BEST1**	0.017471
**GUCY1A3**	0.018441
**PLEK**	0.018441
**EMCN**	0.018441
**CLEC14A**	0.018441
**ENC1**	0.019457
**TMTC2**	0.02052
**ADAM9**	0.021631
**C10orf10**	0.022794
**CFI**	0.024008

**APLNR**	0.024008
**KYNU**	0.024008
**PRKD1**	0.025276
**MFNG**	0.0266
**TYROBP**	0.027982
**MCTP1**	0.029423
**PPARG**	0.029423
**SLC29A1**	0.030924
**EPB41L4A**	0.030924
**TMEM119**	0.032489
**GALNTL4**	0.032489
**GLT25D1**	0.032489
**TSPAN18**	0.032489
**LYVE1**	0.032489
**SKAP2**	0.034118
**ENTPD1**	0.034118
**SLC5A4**	0.035814
**COL15A1**	0.035814
**NAV3**	0.037579
**NOSTRIN**	0.039413
**AOC3**	0.04132
**DDIT3**	0.04132
**IL6**	0.043301
**SEMA4C**	0.043301
**GRAP**	0.043301
**CCL18**	0.045359
**RGS16**	0.047494
**RPS6KA2**	0.049708
**VCAM1**	0.049708
**KCTD12**	0.049708
**MCC**	0.049708
**ARRB1**	0.049708
ADAMTS12	0.054385
STAB1	0.054385
TLR4	0.054385
GPM6B	0.05685
ICA1	0.05685
C10orf11	0.05685
VWF	0.062044
CD14	0.062044
HSPA6	0.064776
TMEM185A	0.064776
PCDH1	0.064776
ABCB4	0.0676
LFNG	0.070519
CTH	0.072008
APOL4	0.079858
FAP	0.079858
NDST1	0.08317
STEAP4	0.086585
GALNT14	0.090103
C2	0.090103
CHN1	0.097456
ACVRL1	0.109297
VPREB3	0.115584
RASSF2	0.117745
TNFSF10	0.117745

DOCK2	0.122138
KLF7	0.122138
TDO2	0.122138
CDH2	0.126644
LRP11	0.136002
ANO2	0.136002
LAIR1	0.140854
ZNF238	0.150907
TTYH3	0.156108
FXYD5	0.156108
C7orf53	0.161426
CTSC	0.166861
ANKDD1A	0.172412
ICAM1	0.183864
HMGB3	0.189764
RAPGEF3	0.22097
KIF21B	0.227546
DCLK1	0.241024
ST6GALNAC3	0.241024
DOCK11	0.254924
C1GALT1	0.254924
FAM65A	0.276534
NLN	0.283932
MRAS	0.299006
CDC42EP1	0.299006
NBPF3	0.314434
HDAC7	0.322273
ENTPD6	0.330192
WWC1	0.354397
FLJ42220	0.396032
MTSS1	0.464987
PBLD	0.473726
PDE2A	0.491236
EMR2	0.535013
OR13C9	0.552435
C21orf122	0.578369
FLJ39653	0.653743
NFASC	0.669808
OR51B4	0.677727
TMEM220	0.677727
PPM1F	0.700994
OR52H1	0.723466
ZNF862	0.737972
FBXO15	0.737972
APOBEC3F	0.758976
RIBC1	0.838574
C4orf42	0.854178
FLJ20464	0.854178
GRM2	0.877862
CBLN3	0.929481
HEJ1	0.937956
NKAIN1	0.937956
TET1	0.950292
GSTM2	0.954641
RNF32	0.962421
ZNF839	0.969076
C14orf148	0.972018

ZMYND17	0.972018
FLJ40852	0.982529
ZNF793	0.999221

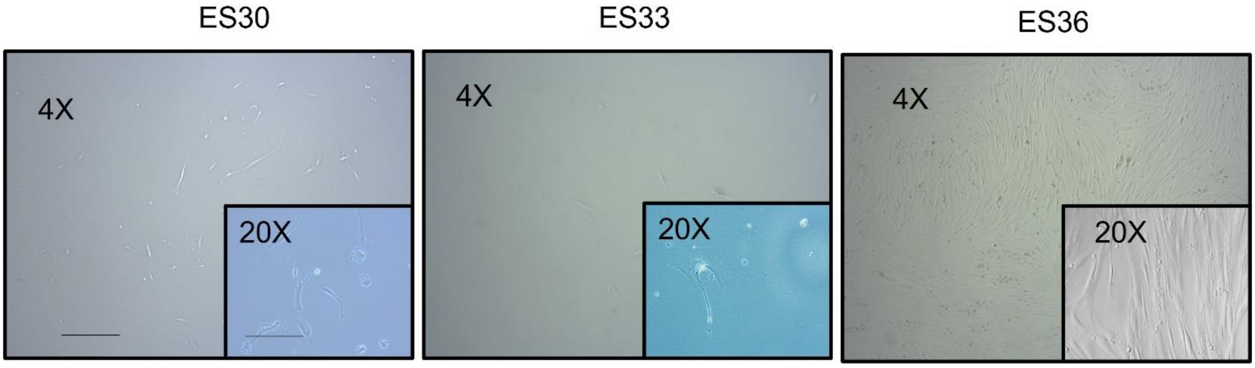

Figure S1. Microscopic analysis of Ewing sarcoma primary cultures ES36, ES33 and ES30. Magnification 100 and 200, Scale 50 and 100μm.

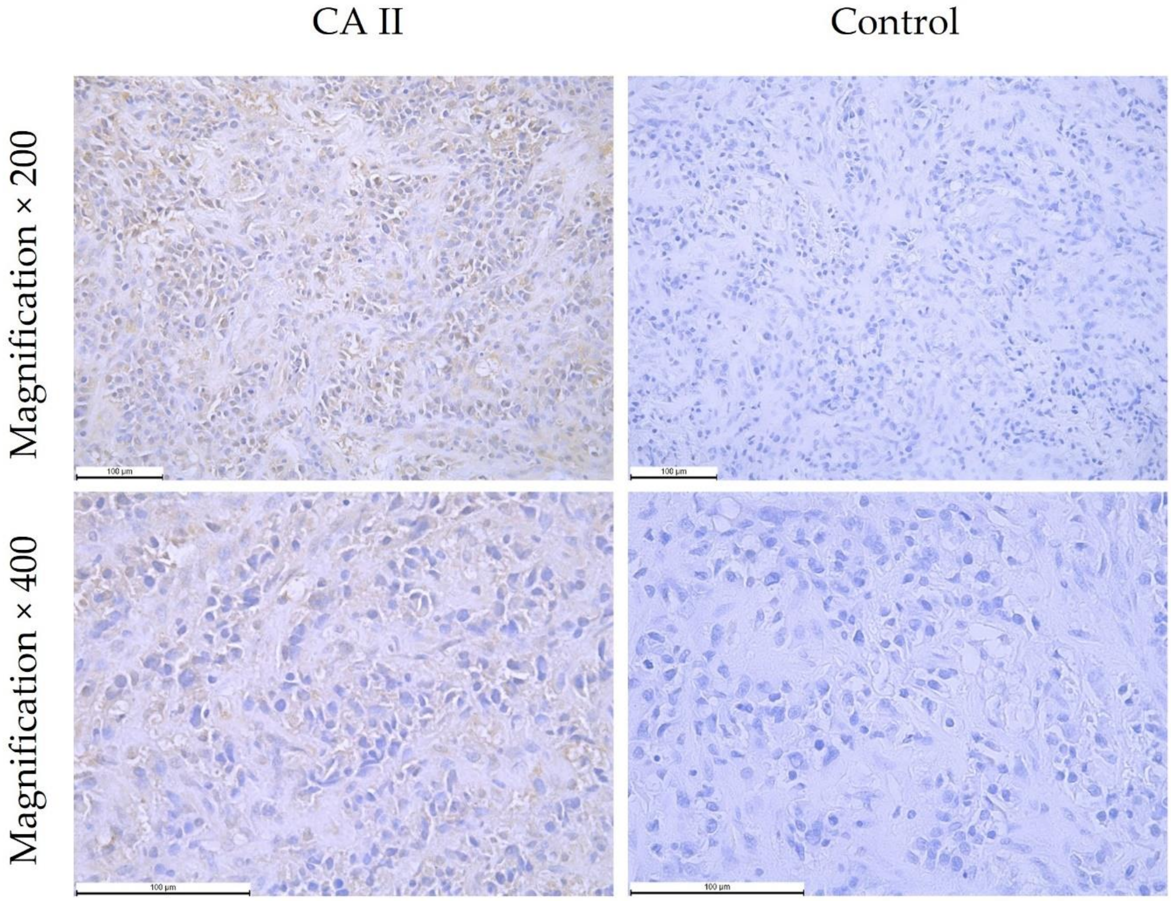

Figure S2. Detection of Carbonic anhydrase type II in the Ewing sarcoma tumor tissue. Slight focal background non-specific staining of stroma and singular erythrocytes. Absence of nuclear or cytoplasmic expression of CA II in cancer cells is detected. Magnification x200 and x400.

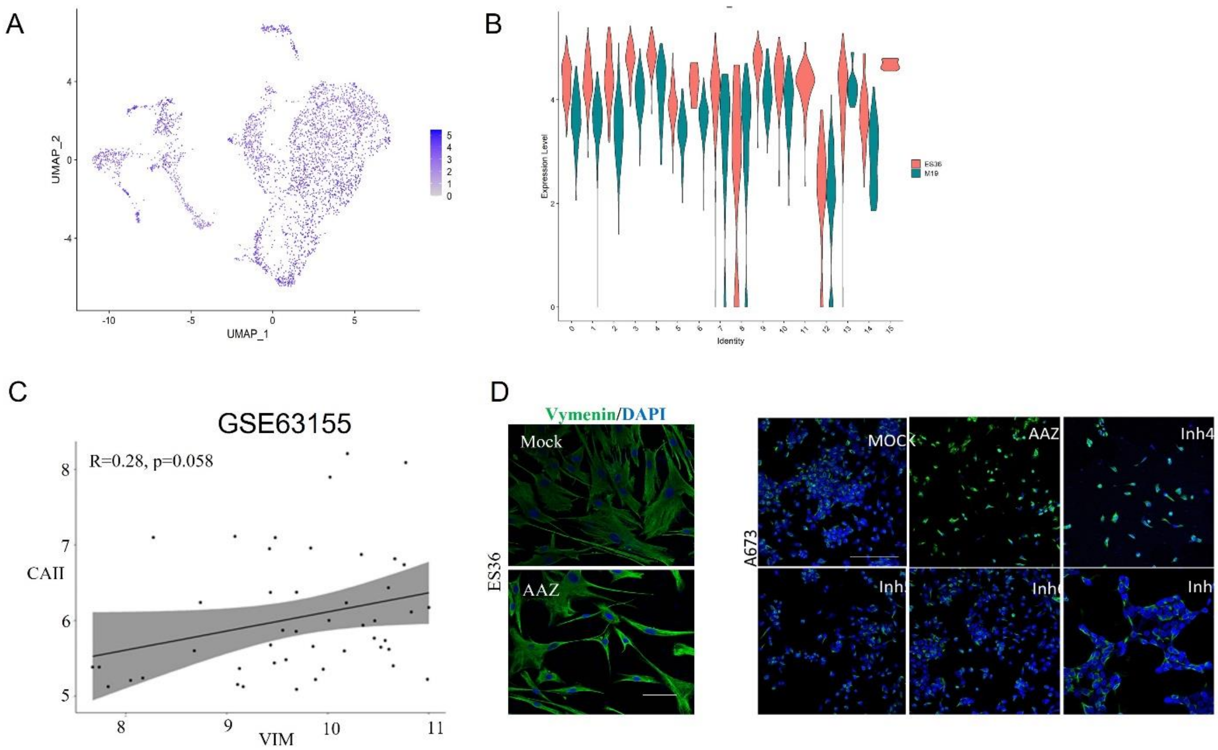

Figure S3. CA II inhibition manifests vimentin reduction in ES sarcoma cells. A)UMAP illustration of cell clustering for vimentin (VIM) expression using dataset (Yakushov S *et al*, 2022); B)Violin clustering shows distribution of vimentin in ES36 primary cellular culture and embryonic fibroblasts M19. Vimentin expression is enriched in cluster 11 of ES36 cells; C)Pearson correlation between CA II and vimentin expression in ES primary tissue. Correlation was done using GSE63155 dataset.R2=0.28; Representative immunofluorescence analysis of vimentin expression during AAZ and Inh4, Inh5, Inh6 and Inh9 treatment of ES36, primary Ewing sarcoma culture and model A673 tumor cells. Nucleus were counterstain with DAPI. Detection of Vimentin was conducted using anti-vimentin antibodies following AlexaFluor 488-labeled anti rabbit secondary antibodies. Images were collected 100x magnification, scale 50 μm.
