# Supplementary material for: Carbonic Anhydrase Inhibitors Induce Ferroptosis through Inhibition of AKT/FTH1 Signaling in Ewing Sarcoma Tumor Cells
Source: Cancers (Basel). 2023 Oct 31;15(21):5225. doi: 10.3390/cancers15215225 (PMC10650537; doi:10.3390/cancers15215225)
Supplement: Supplementary file 1 [file cancers-15-05225-s001.zip › supplementary file S2.pdf]

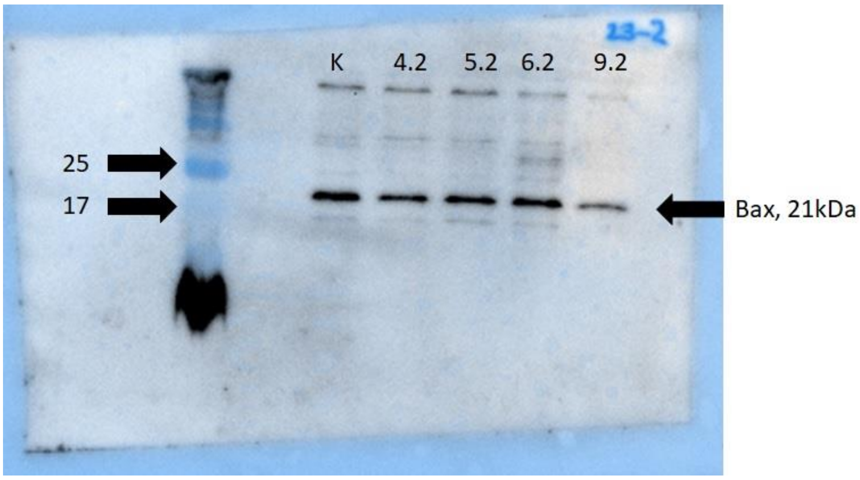

Мембрана 23-9, результат
14.07.
ATG5, FNab00678, 50-55 kDa

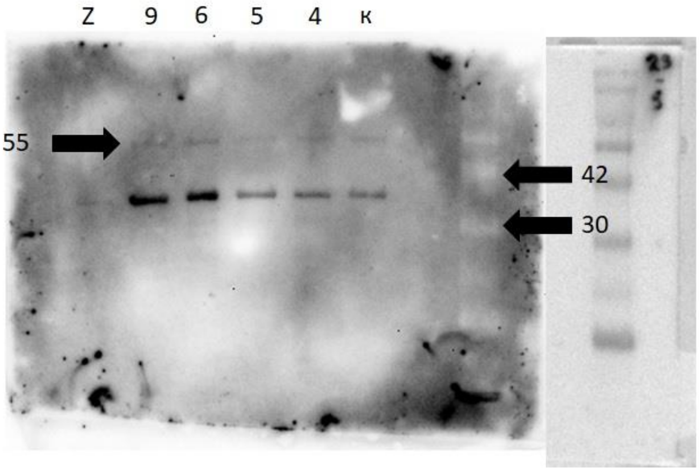

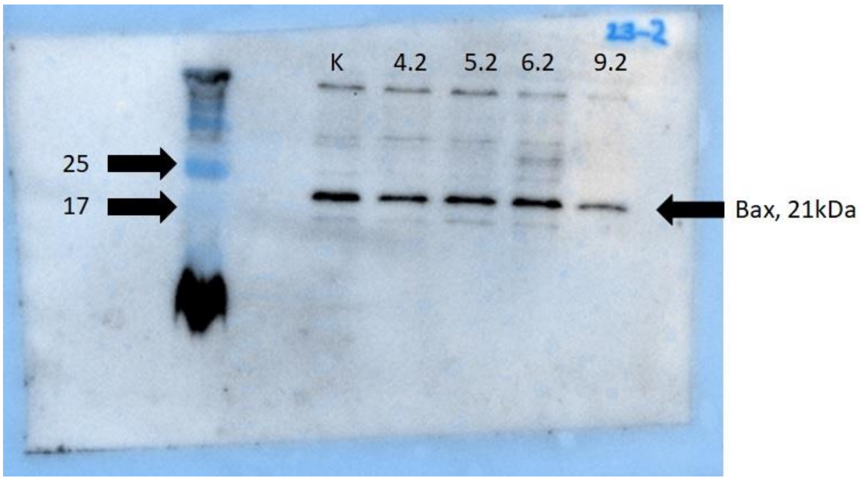

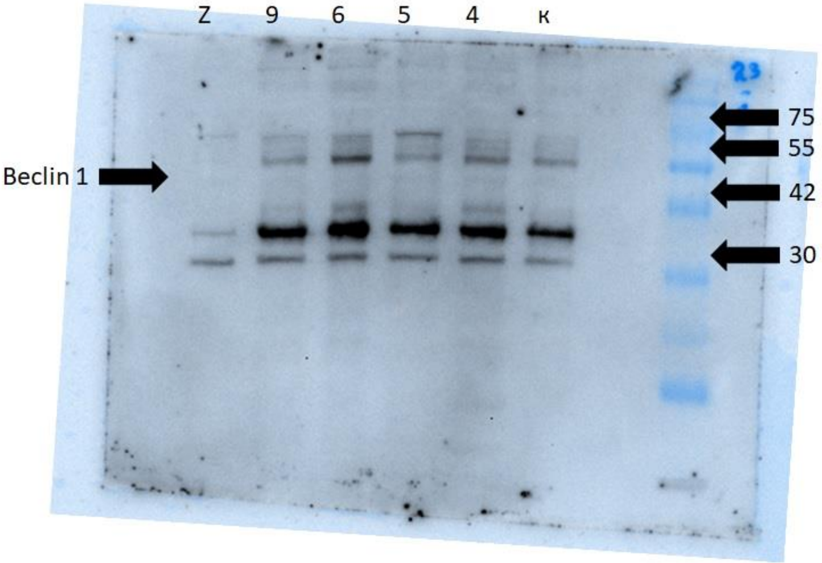
Z
9
6
5
4
к
Beclin 1
75
55
42
30

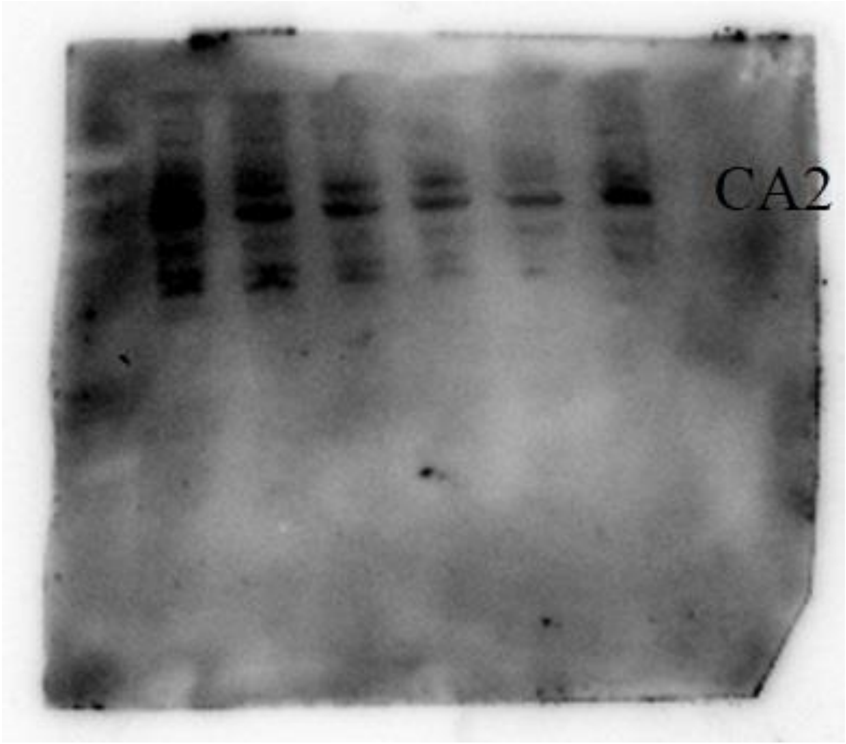
CA2

Мембрана 23-9, результат 13.07.
Cathepsin B, FNab10389, 31/25 kDa

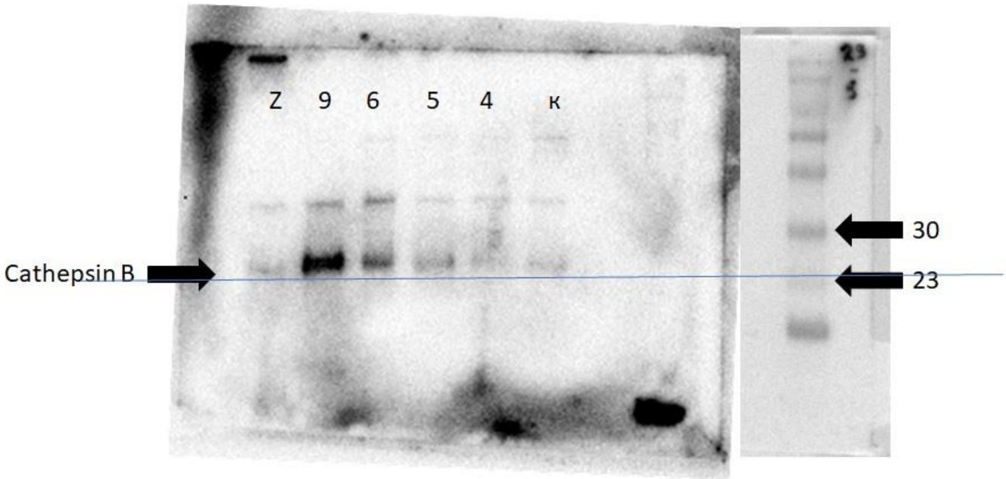

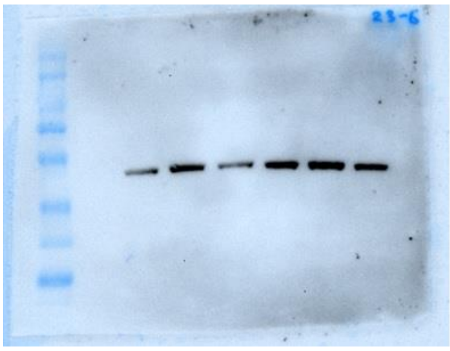

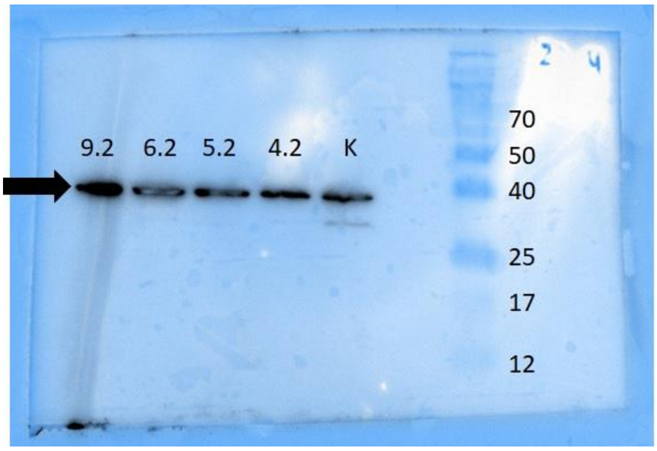

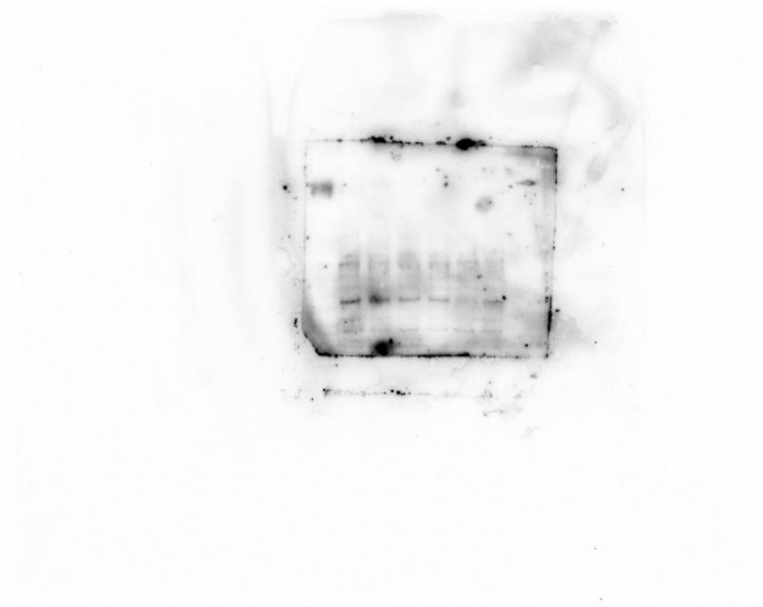

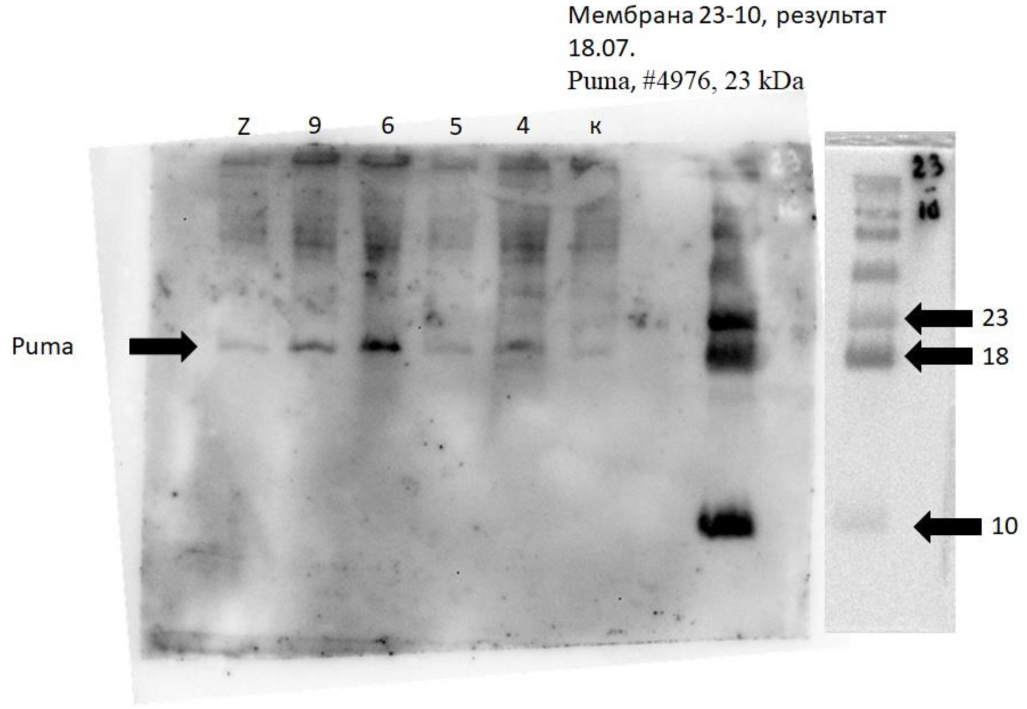

Figure S4: The original blots for Figure 3.

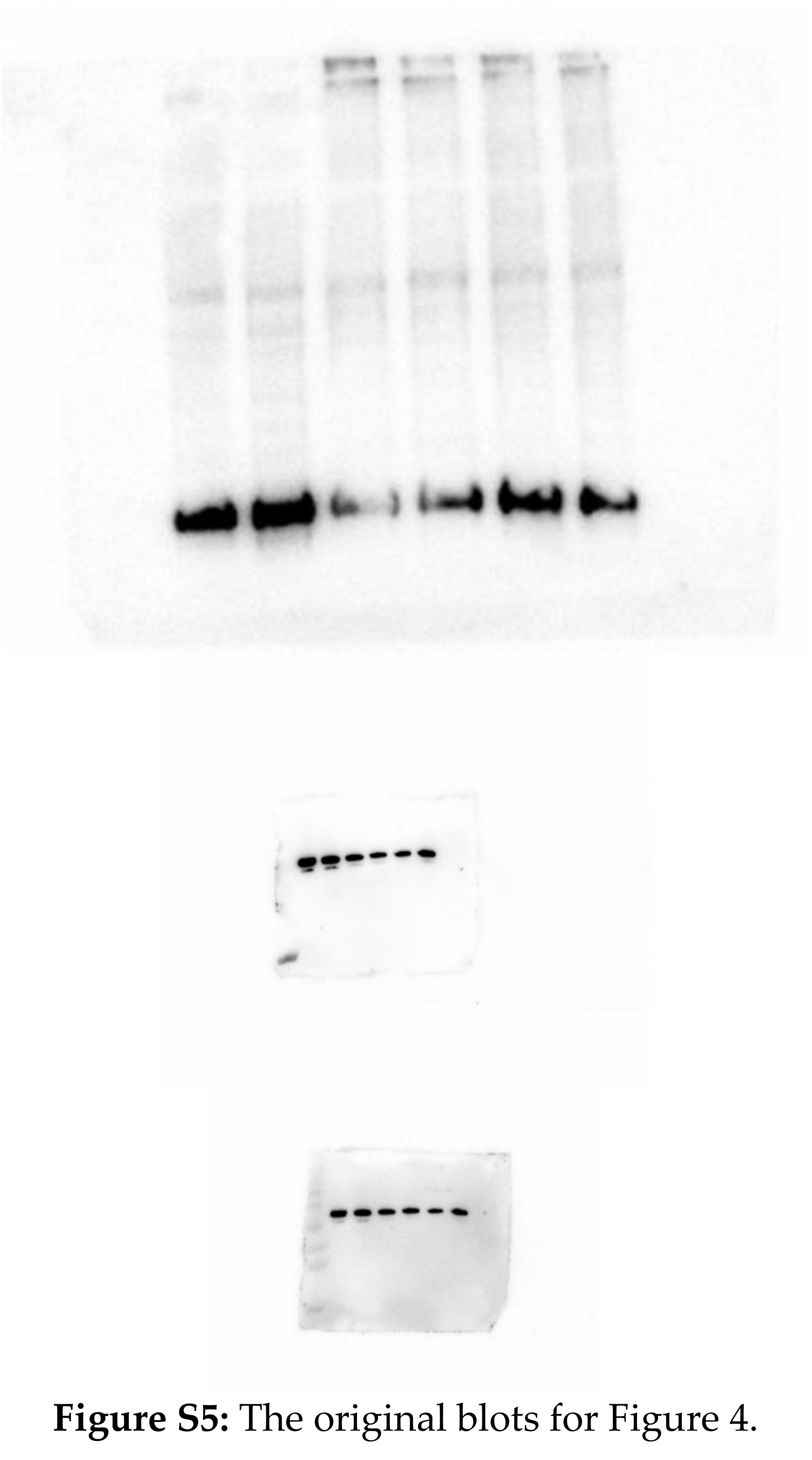

Figure S5: The original blots for Figure 4.
